# Enhancing Thermo‐Osmotic Low‐Grade Heat Recovery by Applying a Negative Pressure to the Feed

**DOI:** 10.1002/gch2.202200238

**Published:** 2023-03-06

**Authors:** Yifan Zhang, Ji Li, Zikang Zhang, Wei Liu, Zhichun Liu

**Affiliations:** ^1^ Department of Engineering Thermophysics School of Energy and Power Engineering Huazhong University of Science and Technology Wuhan 430074 China

**Keywords:** low‐grade heat, negative pressure, numerical simulation, pressure‐retarded membrane distillation, thermo‐osmosis

## Abstract

A newly developed technology, thermo‐osmotic energy conversion (TOEC), is supposed to convert low‐grade heat into power. However, the performance of existing TOEC experiments is deficient. This paper discusses the feasibility of strengthening TOEC by applying negative pressure to the feed liquid, which can reduce air pressure in the membrane pores and molecular diffusion resistance. Theoretical calculation shows that when the cooling and heating temperatures are 40 and 80 °C, respectively, and the transmembrane pressure difference is 5.0 MPa, the TOEC system with a negative pressure of 0.5 bar at the feed side can approach an efficiency of 3.01% and a power density of 16.85 W m^−2^, which increases by 20% and 27% compared with no negative pressure, respectively. Given the nonuniformity in the real system, computational fluid dynamics simulation is used to obtain the correction factor, which is then used to revise the theory prediction results for the first time. Moreover, a lab‐scale experiment also proves that a negative pressure at the feed benefits the performance of the TOEC device. Overall, this research presents a feasible method to enhance a TOEC system, which may promote the development of a more‐efficiently TOEC system for low‐grade heat utilization.

## Graphical abstract

Thermo‐osmotic energy conversion is supposed to convert low‐grade heat into power. The efficiency can be improved by applying negative pressure to the feed liquid. The theoretical calculation, computational fluid dynamics simulation, and experiments are carried out to verify the method.

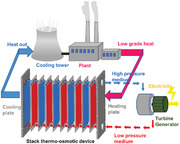

## 1 Introduction

In today's world, energy shortage is becoming increasingly prominent. To solve this problem, besides improving the efficiency of the existing energy facility to save fossil fuels, it is also crucial to develop new, clean, and renewable energy technologies. Factories, nuclear power plants, geotherms, and other sources stably produce low‐grade heat below 80 °C in huge quantities.^[^ ^1^, ^2^, ^3^ ^]^ However, converting low‐grade thermal energy to power effectively, economically, and sustainably is challenging.^[^ ^4^, ^5^, ^6^, ^7^ ^]^ Among the existing low‐grade thermal energy utilization methods, thermo‐osmotic energy conversion (TOEC) is supposed to be a technology with great potential.^[^ ^8^ ^]^ It is also known as pressure‐retarded membrane distillation. Rahimi et al.^[^ ^9^ ^]^ compared several liquid‐based low‐grade thermal energy conversion techniques, including reverse electrodialysis, thermo‐electrochemical cell, TOEC, thermally regenerative electrochemical cycle, and thermally regeneration battery. They concluded that the theoretical efficiency of TOEC can be among the highest among those techniques. Additionally, the power density of the fully optimized stack TOEC system can exceed 100 W m^−2^, with an efficiency of 0.2% (Figure S5, Supporting Information).

As early as the beginning of the last century, the core principle of TOEC technology–thermo‐osmosis effect, was observed by Lippmann in experiments. It is a temperature difference‐driven mass‐transfer phenomenon across the membrane.^[^ ^10^, ^11^ ^]^ It was once seen as an efficient heat engine.^[^ ^12^ ^]^ Recent years have seen the development of TOEC technology, which produces electrical power using low‐grade heat.^[^ ^13^, ^14^ ^]^ In TOEC research, many researchers utilized the hydrophobic membrane to obtain a high mass and power density, which allows vapor to pass through. Furthermore, water is usually selected as the working fluid in lab‐scale experiments for its accessibility, safety, and high surface tension.^[^ ^8^, ^10^, ^15^ ^]^ The process can involve many types of membranes and working fluids, but only hydrophobic membranes and water have been tested thus far. For example, methylene chloride and chloroform may perform better due to better volatility and lower vaporization enthalpy, which needs further study.^[^ ^10^ ^]^ In this study, if there is no other explanation, the membrane is hydrophobic, and the working fluid will likewise be water.

**Figure 1:**
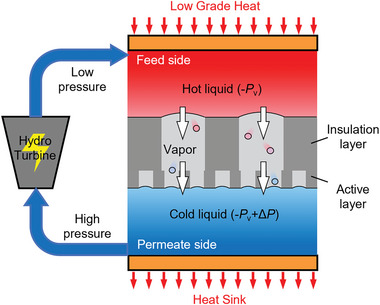
Schematic diagram of the negative pressure thermo‐osmotic energy conversion (NP‐TOEC) system. The working fluid on the feed side has a high temperature and a vacuum pressure (−*P*_v_). It evaporates across the membrane and condenses into the liquid with high hydraulic pressure on the permeate side. The pressure difference (Δ*P*) between the permeate side and feed side can be used to drive the turbine to generate electricity. The negative pressure discussed below exists in the feed chamber, aiming at reducing the molecular diffusion resistance across the membrane.

**Figure** **1** presents the working principle of a TOEC system, which mainly consists of a hydrophobic membrane, two liquid chambers, a heat source, a cold source, and a hydraulic turbine. The higher temperature on the feed side causes the vapor pressure of the working fluid to be greater than that on the permeate side, which generates the force for transmembrane permeation. The working fluid first evaporates into vapor at the interface between the feed liquid and the membrane. After, the vapor across the membrane condenses under high hydraulic pressure into liquid at the membrane–permeate liquid interface. By employing a hydraulic turbine, we can get power from the permeate liquid, and the power density *W* is^[^ ^14^ ^]^

(1) $$\begin{array}{c} W\;=\;\Delta P\cdot J \end{array}$$

where Δ*P* is the operating pressure, the pressure difference between the feed and permeate sides, and *J* is the mass flux. Pressure delay retarded membrane distillation (PRMD) is one of the variants of the TOEC. It was first developed from direct contact membrane distillation (DCMD) based on a liquid and air‐gap hydrophobic membrane system.^[^ ^16^, ^17^, ^18^ ^]^ The PRMD can produce water and power simultaneously.^[^ ^16^, ^17^, ^19^ ^]^ However, the TOEC can only generate electricity because the TOEC is a completely closed system, and the permeate stream is mixed with the feed stream to make the power generation cycle.

From the view of force, the hydraulic pressure on the permeate side comes from the meniscus pressure of the membrane–permeate interface. According to the Young–Laplace formula, the membrane needs to be hydrophobic and have a small pore size to generate a sizeable hydraulic pressure without wetting.

(2) $$\begin{array}{c} \Delta P=-\gamma \left(\frac{1}{R_{1}}+\frac{1}{R_{2}}\right) \end{array}$$

where γ is the surface tension, and *R*_1_ and *R*_2_ are the principal radii of curvature. From the view of thermodynamics, the highest transmembrane hydraulic pressure difference, *P*_TO_, is also limited by the transmembrane temperature. According to the Antoine and Kelvin equations, it can be deduced that *P*_TO_ is^[^ ^14^ ^]^

(3) $$\begin{array}{c} P_{\text{TO}}=\frac{H_{M}}{V_{M}}\left(1-\frac{T_{m,c}}{T_{m,h}}\right) \end{array}$$

where *H*_M_ reflects the transmembrane heat transport caused by the transmembrane mass transfer, that is, the molar enthalpy changes of the fluid entering and leaving the membrane. *V*_M_ is the molar volume of the liquid. *T*_m,f_, and *T*_m,p_ are the surface temperatures on both sides of the membrane. For the case of water across an air‐gap porous membrane, *H*_M_ is the vaporization enthalpy of water. According to Equation (3), even a small temperature differential can result in a significant *P*_TO_.^[^ ^10^, ^14^ ^]^ For instance, a pressure difference of 6.76 MPa can be produced by a water temperature differential of 1 °C at a reference temperature of 60 °C. So, the pore size rather than thermodynamics often limits the operating pressure.

Small pore size and high operating pressure are necessary to achieve high power density. Shaulsky et al.^[^ ^15^ ^]^ used vapor deposition treatment to create hydrophobic mixed cellulose ester flat‐sheet ultrafiltration membranes with nominal pore diameters of 25 and 50 nm, which can resist hydraulic pressure of 2.4 and 4.2 MPa, respectively. A smaller pore size, however, invariably results in a higher mass transfer resistance. Straub et al.^[^ ^8^ ^]^ proposed that one of the critical characteristics of a high‐performing membrane is the asymmetric structure consisting of a layer with tiny pores and a layer with large pores. Moreover, to resist the transmembrane pressure difference, the TOEC membrane should also possess robust mechanical qualities.^[^ ^8^, ^15^ ^]^ Kuipers et al.^[^ ^13^ ^]^ reported that a membrane could keep performance at the operating pressure of 1.7 MPa, while some membranes with a high porosity may face flux reduction under operating pressure.^[^ ^20^, ^21^ ^]^ Besides membrane structure, system optimization is crucial for improving low‐grade thermal energy use. An external regenerator‐equipped TOEC system that can fully use sensible heat was proposed by Straub et al.,^[^ ^8^ ^]^ whose maximum efficiency can theoretically approach 4.1%. However, this system still has severe temperature polarization and would face the challenges of flow control and thermal insulation when applied. Li et al.^[^ ^10^, ^19^ ^]^ proposed the stack TOEC system that utilizes thermal energy stage by stage for efficiency improvement. This system structure has advantages, including low‐temperature polarization, less pump power loss, and good feasibility.

Since research on TOEC systems has not been carried out for a long time, the efficiency in actual experiments is still relatively low, failing to exceed 0.1%.^[^ ^10^, ^14^, ^17^, ^21^, ^22^ ^]^ To increase the efficiency of the TOEC system, new techniques must be researched. For the DCMD technique, several researchers reported that applying negative pressure to the system can increase the transmembrane flux.^[^ ^23^, ^24^, ^25^ ^]^ Liu et al.^[^ ^26^ ^]^ found that negative pressure positively impacts the anti‐pollution and the flow flux in the DCMD system. The results showed that the negative pressure could reduce the membrane's vapor resistance and improve the convective heat transfer coefficient between the liquid and the membrane. This study discusses the feasibility of enhancing TOEC by applying negative pressure to the feed, reducing the air pressure in the membrane pores, and strengthening the transmembrane flux. Based on earlier studies,^[^ ^8^, ^10^, ^14^, ^17^, ^27^ ^]^ a theoretical model of a stack TOEC system with negative pressure is constructed to discuss the strengthening effect. Furthermore, because the heat and mass transfer is uneven in actual conditions, a correction factor was obtained through computational fluid dynamics (CFD) simulation to correct the theoretical results. Moreover, experiments are conducted on the TOEC system with a single stage. The strengthening effect is seen in experiments, and numerous significant components are investigated. This job will inspire the relevant researcher to improve the TOEC system.

## 2 Results and Discussion

### 2.1 Characterization of Membranes

A two‐layer membrane is used in theory prediction. Its thin functional layer features pores that are 20 nm in diameter and 10 µm thick, which can tolerate a sizeable hydraulic pressure on the permeate side without wetting. Its thick insulation layer features pores that are 500 nm in diameter and 300 µm thick, so it can enhance the overall mechanical strength and reduce transmembrane heat conduction loss. The membrane's overall permeability coefficient is ≈2.86 × 10^−7^ kg/(Pa s m^2^) (Calculated according to Section S1, Supporting Information).

**Figure 2:**
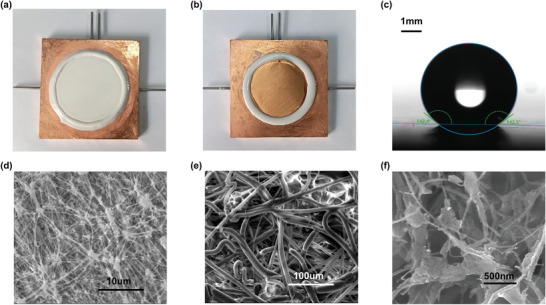
a) Image of the working plate on the permeating side. The membrane has an effective working area of around 18 cm^2^. b) Image of the working plate on the feed side. The brass woven mesh supports the membrane and improves the water supply. c) The water contact angle on the membrane surface is 143.0 ± 0.9°. d,e) Images of the active PTFE layer and the support PP layer under an environmental scanning electron microscope. f) Image of the active PTFE layer under field emission scanning electron microscope.

Similarly, a commercial composite hydrophobic membrane is employed in the experiment, and it has a structure with three layers. As shown in **Figure** **2**, the outer two functional layers are made of polytetrafluoroethylene (PTFE). It is hydrophobic with a water contact angle of 143.0 ± 0.9° and has a small pore diameter of 100 nm. The hydrophobic microporous polypropylene (PP) insulation layer in the center has a much larger pore diameter, ≈20 µm. The silicone rubber is used to adhere the membrane to the permeate‐side plate, and a brass wire mesh is added to the feed‐side plate to provide an auxiliary liquid supply. **Table** **1** and Table S1, Supporting Information, display the individual performance parameters for membranes.

**Table 1:** Characteristics of the membranes for the experiment and prediction

	Prediction		Experiment	
	Functional layer	Insulation layer	Functional layer	Insulation layer
Nominal pore diameter, nm	20	500	100	2000
Thickness, µm	10	300	20 ± 6	242 ± 8
Porosity	0.70	0.70	0.854 ± 0.028	0.605 ± 0.014
Tortuosity^a)^	1.20	1.20	1.08	1.29

^a)^ Determined by τ = ε^−0.5^.

### 2.2 Theoretical Prediction

**Figure 3:**
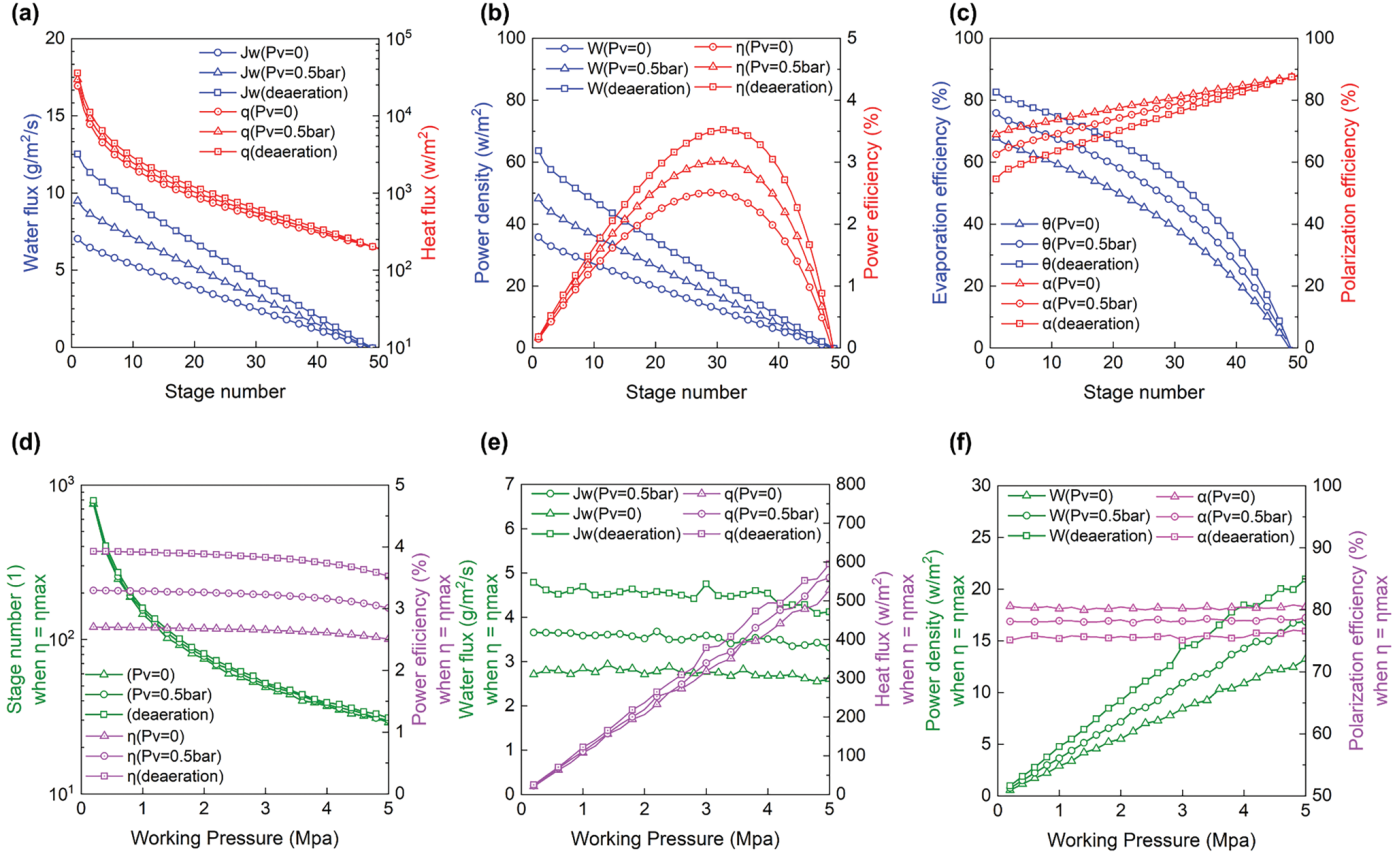
Theoretical performance of the stack NP‐TOEC system between 40 and 80 °C. a–c) Performance with the total number of working stages under three different conditions while keeping the operating pressure *P*_w_ at 5 MPa. d) The required stage number with other operating pressures when the power efficiency maintains the maximum under three different conditions. e,f) Performance with the total number of working stages under three conditions while maximizing power efficiency.

The theoretical calculation is valuable and effective for the TOEC system. In **Figure** **3**, the conditions of no vacuum pressure (*P*_v_ = 0), a vacuum pressure of 0.5 bar (*P*_v_ = 0.5 bar), and complete deaeration on the feed side are compared. The operating pressure and the temperatures of the cold and heat sources remain constant to study the impact of stage numbers. Unlike many other TOEC systems or PRMD systems in the literature, as shown in Figure S2, Supporting Information, the TOEC system in this study does not require a pump to drive, so the pump power consumption within the TOEC system does not need to be considered. Meanwhile, the system's cold and hot plates only involve energy transfer, not mass transfer. Therefore, if the heat loss caused by the temperature difference is to be analyzed, the cold and hot plates can be taken out separately for decoupling analysis. Since various heat exchangers can be adapted for cold and hot plates, their efficiency is brutal to determine directly. If it is assumed that the energy transfer efficiency on the cold and hot plate sides are η_c_ and η_h_, respectively, and the turbine power generation efficiency is η_turbine_. In that case, the total system efficiency η_total_ can be calculated as:

(4) $$\begin{array}{c} \eta_{\text{total}}=\eta_{\text{TOEC}}\times \eta_{c}\times \eta_{h}\times \eta_{\text{turbine}} \end{array}$$

In addition, it is worth noting that the cooling temperature is set at a relatively high 40 °C, and the heat flux of the system is also very low, enabling the cold source to dissipate heat without any power dissipation through the passive heat exchanger. The power density calculation in this experiment can reflect the system's ability to operate and generate electricity. So power efficiency is defined as output power density divided by input heat flux.

It has been discovered that deaeration or negative pressure on the feed side can significantly increase the transmembrane water flux. As shown in Figure 3b, the power density (total water flux multiply operating pressure) and power efficiency is significantly increased because the output power density is a function of the working pressure and total water flux. The heat flux decreases exponentially due to better thermal power utilization with more stages. However, the temperature difference across each stage will linearly decrease if there are more stages, leading to the lack of driving force for transmembrane permeation. In summary, the power density decreases linearly with the stage number while the power efficiency rises and descends.

When applying negative pressure to the feed side, the TOEC system has a higher efficiency at a certain stage number. Because the partial pressure of water remains constant, the total pressure within the membrane decreases, causing the partial pressure of air to lower. Consequently, the molecular diffusion decreases resistance, and the water vapor flux increases. In the group *P*_v_ = 0, the best work‐power efficiency of 2.51% is achieved at the working stage of 29, and the corresponding power density is 13.25 W m^−2^. While in the group *P*_v_ = 0.5 bar, the maximum power efficiency is increased to 3.01% with 30 working stages, corresponding to a power density of 16.85 W m^−2^.

The viscous term is considered in the deaeration group except for molecular diffusion. Compared with the group *P*_v_ = 0.5 bar, the highest power efficiency of the deaeration group is improved by ≈17%. It can approach a power efficiency of 3.52% and a power density of 20.99 W m^−2^ with 31 working stages. An electricity generation efficiency of around 3.5% does not make it high. However, it is 31.4% of the relevant Carnot efficiency. Viscous transport is typically considered negligible for the TOEC process. Because some noncondensable gas (like air) is trapped in the membrane, which maintains the total pressure within the membrane near constant, the viscous flow lacks transport power. In the ideal case of sufficient degassing, the partial pressure of air within the membranes dramatically decreases. On the one hand, the molecular diffusion resistance decreases, favoring the Knudsen resistance. On the other hand, the driving force for viscous flow increases dramatically because the total pressure gradient across the membrane reappears in the absence of stagnant air. So, the transmembrane flow is considered to fall within the Knudsen‐viscous transition region.^[^ ^28^ ^]^

Other parameters, such as evaporation efficiency α and temperature polarization coefficient θ, must be considered in addition to power density and power efficiency.^[^ ^10^ ^]^

(5) $$\begin{array}{c} \alpha =\frac{1}{n}\cdot \frac{J_{W}\Delta H_{\text{vap}}}{q} \end{array}$$

(6) $$\begin{array}{c} \theta =\frac{\sum_{i=1}^{N}\left(T_{m,F}^{i}-T_{m,P}^{i}\right)}{T_{h}-T_{c}} \end{array}$$

where *J*_W_ is the transmembrane mass transfer flux and Δ*H*_vap_ is the enthalpy of vaporization. *i* and *n* are the current and total working stage numbers, and *q* is the heat flux. $T_{m,F}^{i}$ and $T_{m,P}^{i}$ are the temperature of the membrane surface on the feed side and permeate side. *T*_c_ and *T*_h_ are the cooling and heating temperature.

As shown in Figure 3a–c, the heat flux will increase simultaneously after applying negative pressure. Moreover, the heat conduction loss will be more significant within the working plates and the liquid region. Therefore, the temperature polarization coefficient decreases. The smaller the temperature polarization coefficient, the smaller the temperature difference across the membrane, which limits further improvement of the TOEC system performance. A similar phenomenon was also found and analyzed in membrane distillation.^[^ ^29^ ^]^ To solve this problem, in one way, the working plate should be thinner and add porous high thermal conductivity materials within the liquid region. On the other way, we may need to decrease the membrane's thermal conductivity, such as using a thicker membrane, so a large temperature difference will be maintained between its two surfaces. Nevertheless, a thicker membrane will typically be bad for water vapor transport. Luckily, it is worth mentioning that the mass transfer coefficient of the viscous flow term is proportional to the square of the pore diameter. Suppose the insulation layer has a larger pore diameter. In that case, the mass transfer coefficient of the viscous term will be significantly higher than that of the Knudsen term, and its mass transfer resistance will decrease drastically. In this way, the functional layer with a small pore diameter dominates the mass transfer resistance of the whole. Therefore, the thickness of the insulation layer can be appropriately increased to reduce the heat conduction loss without too much influencing the mass transfer resistance. This requires different membrane structure design to improve the water flux and enhance its pressure‐bearing capacity (Section S4, Supporting Information).

Figure 3d shows the required working stage number for the maximal efficiency under different pressure conditions. Figure 3e,f shows the system performances with pressure conditions while the working stage number is set under the maximal efficiency. It can be seen that, even at low operating pressure, high efficiency can still be achieved by significantly increasing the stage number. However, a large working stage number will reduce the system's heat flux and power density. And in the actual device, too many working stages will increase the investment and enhance the heat leakage to the environment. Considering the membrane deformation, the pore size and porosity of the functional layer decrease under high working pressure, leading to an increase in mass transfer resistance and a reduction in water flux and power efficiency compared to lower working pressure. Increasing the operating pressure to operate the TOEC system more economically is very important. Furthermore, the membrane's mechanical stability should be strengthened to minimize membrane deformation. Similar to the trend in Figure 3a–c, under the same operating pressure, the efficiency and power density of the system can increase a lot after applying negative pressure to the feed. In the meantime, working stage numbers increase slightly. So, the negative pressure on the feed can improve the TOEC system performance under various operating pressure.

### 2.3 CFD Simulation

**Figure 4:**
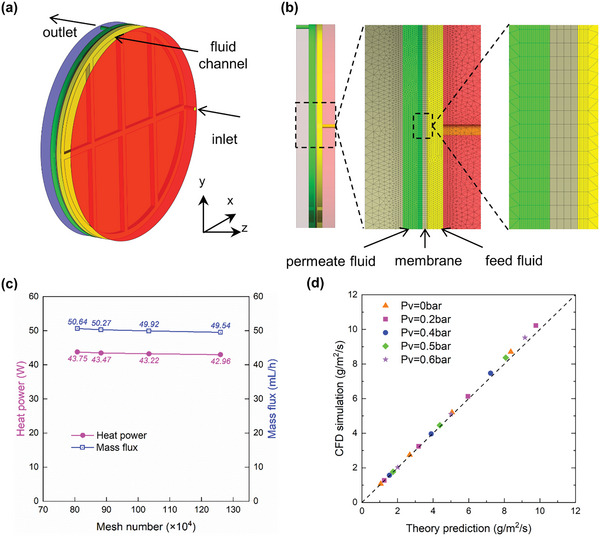
a) The geometric model of the lab‐scale TOEC device. b) The mesh structure from the front view of the TOEC device. c) The grid independence verification. d) The CFD simulation water flux compared with the theoretical prediction value at different feed‐side vacuum pressures.

The CFD simulation and theoretical prediction are carried out under the same condition. The parameter details are the same as in the Experiment column of Table 1 and Table S1, Supporting Information. **Figure** **4**a,b shows the geometry model and mesh model in simulation. Both the inlet and outlet are pressure boundary conditions, and the inlet temperature is 25 °C (consistent with the experiment). By altering the gauge pressure at the inlet and outlet, it is possible to simulate negative pressure on the feed side and high pressure on the permeate side. Constant temperature boundary conditions are employed for the heating and cooling surfaces according to the experimental value. A hexahedral mesh is adopted in the liquid chamber close to the membrane surface, and the mesh thickness is only 0.02 mm. More details about grid independence verification and governing equations can be seen in Section S3, Supporting Information.

The theoretical prediction is ideal, efficient, and 1D, which cannot reflect the impact of uneven fluid channels. On the contrary, CFD simulation can show the nonuniformity of heat and mass transfer. However, it is time‐consuming, especially for some complicated structures. A correction factor can be calculated compared with the 3D CFD results with the theory prediction. With this correction factor, we can use the corrected theory prediction model to reflect the real overall performance. It will be more precise and still has highly efficient. As shown in Figure 4c, four different sets of meshes have been simulated, corresponding to the fluid gap grid thickness of 25, 20, 15, and 10 µm, respectively. The scheme of 883 714‐grid was chosen for regular simulation, whose heat power and mass flux errors are less than 1.2% and 1.5%, compared with the 1 259 619‐grid strategy. Figure 4d shows the comparison of water flux between the CFD simulation and the theoretical prediction. With a correction factor of 0.9, all data points are almost evenly distributed on the diagonal, indicating that the corrected theoretical prediction model matches the CFD simulation results well. The performance of a natural system can be predicted more conveniently using this correction factor.

**Figure 5:**
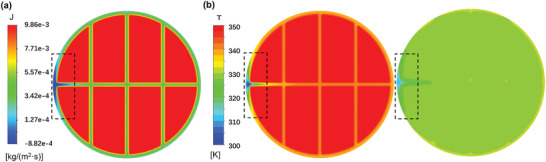
a) The water flux contour at the fluid‐membrane interface. b) Temperature contours at two fluid‐membrane interfaces at the feed side and permeate side.

One of the advantages of CFD simulation is that it can easily obtain the internal distribution of each physical quantity, which is difficult to obtain from experiment or theory calculation. As shown in **Figure** **5**a,b, the mass transfer flux near the channel region is significantly smaller than the flux in the other region due to the thicker liquid gap. Moreover, beyond expectations, there is a negative mass flux near the inlet region. The temperature of the inlet water is 25 °C. After insufficient heating, its temperature is still lower than the cold source, resulting in a negative temperature gradient in the inlet region. And a negative temperature gradient leads to a negative local vapor pressure difference. Therefore, the water flux is negative. However, due to the tiny transmembrane temperature difference at the inlet region, the negative water flux is small, only ≈10% of the average flux. So, the negative flux at the inlet region has little influence on the total performance. While the channel region, whose area accounts for ≈20%, dominates the water flux difference between the original theoretical prediction value and the CFD simulation results.

### 2.4 Experimental Results

**Figure 6:**
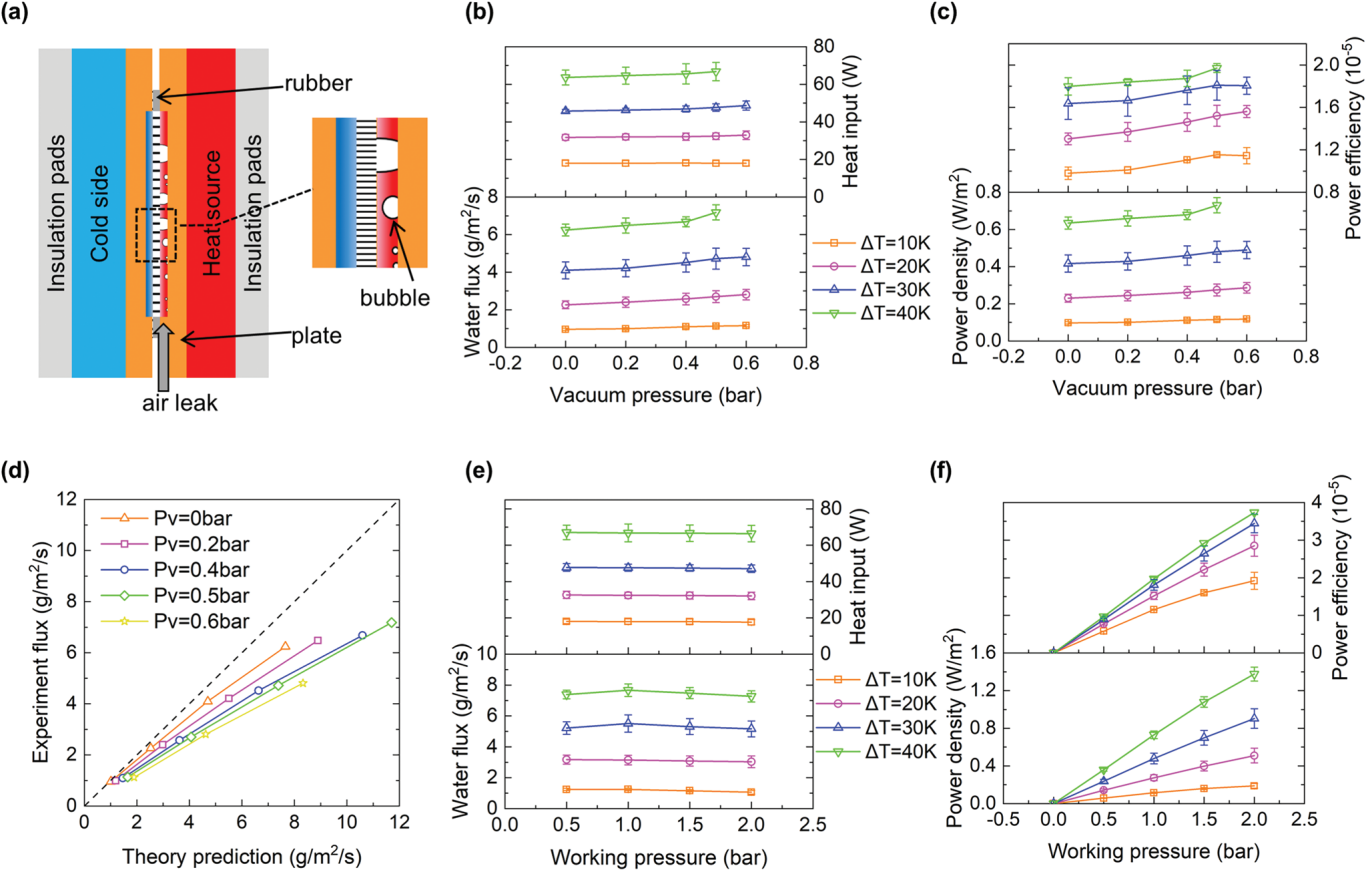
a) Schematic of the lab‐scale single‐stage experimental device and air leakage phenomenon. b,c) Performances of the NP‐TOEC system under various feed side vacuum conditions, with the operating pressure *P*_w_, held constant at 1.0 bar. The heat input represents the total thermal energy input, whereas the water flux and power density are standardized to the working membrane area. d) When *P*_w_ = 1.0 bar, the experimental water flux is compared with the theoretical prediction value under different *P*_V_. e,f) Performances of the NP‐TOEC system at various operating pressures, with the vacuum pressure *P*_V_, held constant at 0.5 bar at the feed side. Error bar: standard deviation.

A TOEC experiment with a single working stage was carried out to verify the enhancement effect of negative pressure. **Figure** **6**a shows the lab‐scale single‐stage experimental device. In Figure 6b,c, the negative pressure on the feed side is different from 0 to 0.6 bar, where the operating pressure keeps at 1.0 bar. The water flux increases with negative pressure, consistent with the theory prediction. At the heating temperature of 60 °C, compared with no negative pressure, the system's water flux and power efficiency are increased by ≈24.0% and 19.7%, respectively, when the feed is applied to a 0.6 bar negative pressure. The power density and thermal power efficiency positively relate to the negative pressure. Because under the same operating pressure, the water flux increases, and the heating power does not change much. At the same time, it shows that a larger temperature difference between the cold and heat sources can effectively improve the system's operating performance. A larger temperature difference can generate a more considerable vapor partial pressure difference across the membrane, which increases the transmembrane driving force. So, the water flux increases with the temperature difference. Furthermore, to improve system performance by improving temperature difference, it can choose a heat source with a higher temperature and employ the membrane with better thermal endurance. This is achievable in practice, as a large amount of low‐grade thermal energy can reach 90 °C or higher,^[^ ^3^ ^]^ and materials like polyethylene terephthalate can tolerate 120 °C long term.

The liquid channel region has a thicker water gap, so the relevant heat transfer resistance is much larger. A correction factor was obtained by comparing the CFD simulation results with the theoretical results to consider this nonuniformity (Section S3, Supporting Information). The CFD simulation is carried out using FLUENT based on the mass‐jump method.^[^ ^30^, ^31^ ^]^ Using this correction factor, the overall average performance of the experimental device is achieved through the theoretical model. However, the experimental results seem to be less than the theoretical prediction value at a high vacuum. As shown in Figure 6d, the lower the vacuum pressure is, the more consistent the results. This should be related to the noncondensable gas in the feed chamber, which hinders heat conduction and mass transfer.

During the experiment, gas bubbles are observed steadily appearing in the gas accumulator, becoming more severe at higher vacuum pressure. Three reasons may explain this phenomenon. First, the feed liquid has some soluble noncondensable gas, which will dissolve in the feed chamber due to the higher temperature. And a low feed pressure makes the effect stronger. Second, silicone rubber is permeable to gas, so air can leak into the feed chamber, and when the vacuum degree increases, the air leakage of the system becomes more serious. Third, because the air has a larger specific volume under negative pressure, the volume of gas within the feed chamber expands more greatly. This is similar to the stagnant air in the air gap membrane distillation system. As shown in Figure 6e,f, the impact of operating pressure is also studied. The working pressure varies, and negative pressure maintains at 0.5 bar. Like the above theoretical prediction, increasing the operating pressure can effectively improve the power efficiency and density of the TOEC system. However, it also causes a slight decrease in the water flux. On the one hand, when the operating pressure is high, local reverse transmembrane leakage may occur in the pores with relatively large sizes. On the other hand, the membrane may deform under a large pressure difference, which might increase flow resistance and impede water supply.

The operating pressure in the experiment is not high because the membrane's pore is not narrow enough. The limited operating pressure results in low energy conversion efficiency at the order of 10^−5^, which is also the result of many other experimental pieces of research. However, the experiment is mainly at verifying the theory prediction. And the performance improvement has been achieved successfully after applying negative pressure at the feed side. Besides, unlike the DCMD system, the stack TOEC system needs no pump to power the water into the feed side, which will save the pump power. With more working stage numbers, the heat flux can decrease a lot. From the above theoretical calculation, when the stage number is 7–33, the heat flux of the degassed system is 5173–588 W m^−2^, which is not very high. So passive heat dissipation methods can be applied to save electricity consumption for cooling, such as the loop heat pipe.^[^ ^32^ ^]^ Besides, recent research on the TOEC system can generate electricity and light the LED via electrokinetic phenomena in the experiment.^[^ ^33^ ^]^ So the stack TOEC system can produce electricity, and applying negative pressure to the feed can enhance it.

## 3 Conclusion

To prove the feasibility of strengthening TOEC by applying negative pressure to the feed, a theoretical prediction model for NP‐TOEC is developed and corrected with CFD simulation results. And relative experiments are also conducted for verification. Theoretical calculation shows that when the operating pressure is 5.0 MPa, the negative pressure on the feed side is 0.5 bar, and the cooling and heating temperature are 40 and 80 °C, the efficiency of the 30‐level system can reach 3.01%, corresponding to a power density of 16.85 W m^−2^, which increases by 20% and 27% compared with no negative pressure, respectively. If the noncondensable gas is completely removed, the transmembrane flow will transform from the Knudsen‐molecular transition region to the Knudsen‐viscous transition region, and the mass transfer resistance will further decrease. A correction factor can be easily obtained using CFD simulation to correct the theoretical prediction results and display a real uneven system's inner physical quantity distribution. In the lab‐scale experiment, a flow rate improvement of ≈24% was observed, proving the benefits of applying negative pressure at the feed. This study offers a workable method to enhance TOEC systems, which may encourage the growth of high‐performance TOEC systems for low‐grade heat utilization.

## 4 Experimental Section

### Theory Prediction Method

Based on previous studies on thermo‐osmosis and membrane distillation,^[^ ^10^, ^14^, ^27^ ^]^ a theoretical prediction model for stack thermo‐osmotic systems was established (Section S1, Supporting Information). Water was employed as the working fluid in the model, which was consistent with the working fluid used in the experiment. Referring to the dusty‐gas model, the prediction model comprehensively considered the Knudsen diffusion term, molecular diffusion term, and viscous flow term to calculate water flux. Antoine formula and Kelvin correction were used to determining the pressure. The functional layer's compression was considered, which cannot be neglected under high pressure.^[^ ^21^, ^34^ ^]^ Due to the low flow rate, convective heat transfer was negligible. The temperature calculations were made on Fourier's law of heat conduction. The theoretical prediction was a 1D hydraulic calculation. However, the heat and mass transfer were uneven in the 2D plane direction, and the overall average performance could be obtained by multiplying a correction factor. The impact of negative pressure on the TOEC system could be investigated by adjusting the pressure of the feed side, and the impact of residual air could be studied by including a viscous flow term or not.

### CFD Simulation Method

CFD simulation was carried out using FLUENT To correct the 1D treatment of the theoretical prediction based on the mass‐jump method.^[^ ^30^, ^31^ ^]^ Fluent and UDF worked together to model how heat and mass moved between TOEC components. In the fluid domain, the first layer grid's temperature and pressure were monitored on the membrane–water interface of both sides, and the mass transfer flux at each grid node was computed using the dusty‐gas model.^[^ ^28^ ^]^ After that, the mass source, momentum source, and energy source terms were calculated and returned to the grids. The membrane itself was set as a solid domain. During the simulation, the physical properties of the membrane that affected its heat transfer performance, such as density, thermal conductivity, and specific heat capacity, were set by the experimental membrane's specifications.

### Experimental Setup

A single‐stage TOEC system was chosen instead of a multistage due to better airtightness and assembly ability. The experiment uses an open cycle to measure the water rate (Section S2, Supporting Information). A pressure gauge and a measuring cylinder measured the water's pressure and flow rate. So the output work could be calculated. Water would be boiled for degassing before the experiment begins. However, there would still be a small amount of air re‐dissolved in the water during the test. Moreover, air leakage happened on the feed side, especially when negative pressure was applied. As a result, a gas accumulator was created and installed at the system's highest point. The gas in the liquid chamber could be successfully discharged using the difference in gas–liquid density, and its unfavorable impacts could be lessened. Using a vacuum pump, the gas accumulator could drain its contents. The power needed for this process was small in comparison to the power generated because noncondensable gases were not very soluble in water.

## Conflict of Interest

The authors declare no conflict of interest.

## Supporting information

Supporting Information

## Data Availability

The data that support the findings of this study are available from the corresponding author upon reasonable request.
