# Supplementary material for: Enhancing Thermo‐Osmotic Low‐Grade Heat Recovery by Applying a Negative Pressure to the Feed
Source: Glob Chall. 2023 Mar 6;7(4):2200238. doi: 10.1002/gch2.202200238 (PMC10069319; doi:10.1002/gch2.202200238)
Supplement: Supplementary file 1 — Supporting Information [file GCH2-7-2200238-s001.pdf]

Global Challenges

Open Access

Supporting Information

for *Global Challenges,* DOI: 10.1002/gch2.202200238

Enhancing Thermo-Osmotic Low-Grade Heat Recovery by Applying a Negative Pressure to the Feed

*Yifan Zhang, Ji Li, Zikang Zhang, Wei Liu, and Zhichun Liu**

Enhancing Thermo-osmotic Low-grade Heat Recovery by Applying a Negative Pressure to the Feed (Supplementary Information)

*Yifan Zhang, Ji Li, Zikang Zhang, Wei Liu, Zhichun Liu**

* Corresponding Author

School of Energy and Power Engineering, Huazhong University of Science and Technology, Wuhan 430074, China.

Email: zcliu@hust.edu.cn

1. Theory prediction details

According to earlier research on TOEC systems [1-4], the transmembrane mass transfer flux J_W can be calculated as the product of the transmembrane working fluid's vapor partial pressure difference and the mass transfer coefficient B_W.[2]

$$J_W = B_W(P_{V,F} - P_{V,P}) \tag{1}$$

where $P_{V,F}$ and $P_{V,P}$ represent the working fluid's partial pressure at the membrane surfaces on the feed side and permeation side, respectively, and B_W represents the transmembrane mass transfer coefficient. Following earlier studies, the working fluid should select a liquid with high surface energy, so it can generate a meniscus to support large hydraulic pressure without wetting. Because water is available, nontoxic, safe, and has high surface energy, it is typically used as the working fluid in the TOEC system. The Dusty-Gas model calculates the B_W, which contains the Knudsen diffusion term (B_K), molecular diffusion term (B_D), and viscous flow term (B_V).[5]

$$B_W = \left(\frac{1}{B_K} + \frac{1}{B_D}\right)^{-1} + B_V \tag{2}$$

The B_K reflects the collision between the water vapor and the membrane pore wall, which is correlated with pore size. The B_D describes the interaction of the working fluid vapor with other noncondensable gas, such as air. It is connected to the partial pressure of noncondensable gas inside. The B_V reflects the interaction between the working fluid vapor. When the TOEC system is inadequately degassed, the air within the membrane keeps the total pressure inside constant. Therefore, the viscous flow cannot be calculated without a driving force. The following formulas are used to compute the mass transfer coefficients.[2]

$$B_K = \frac{2}{3}\frac{\varepsilon r}{\xi\delta}\sqrt{\frac{8M}{\pi RT}} \tag{3}$$

$$B_D = \frac{\varepsilon P D_w M}{\xi\delta P_a RT} \tag{4}$$

$$B_V = 0.125\frac{\varepsilon r^2 M P_m}{\delta\xi R T_m \eta} \tag{5}$$

The membrane's porosity, pore radius, thickness, and pore curvature are denoted in these formulations by ε, r, δ, and ξ, respectively. The working fluid's molar mass and viscosity are M and η, respectively. In addition, P, P_a, and P_m are the total gas pressure, partial pressure, and average pressure within the membrane. R is the ideal gas constant, T is the temperature under the Kelvin temperature scale, and T_m is the average temperature across the membrane. The Antoine formula can be used to determine the partial pressure of the working fluid with Kelvin correction. The saturated vapor pressure P_S for water is:[1]

$$P_S = e^{23.1964 - \frac{3816.44}{T_m - 46.13}} \tag{6}$$

The $P_{V,F}$ and $P_{V,P}$ represent the working fluid's vapor pressure on the feed and permeate sides. They are influenced by negative pressure to the feed side (P_{vac}) and hydraulic pressure to the permeate side (P_w).[1]

$$P_{V,F} = P_{S,F} e^{\frac{P_{vac} V_m}{R T_{m,F}}} \tag{7}$$

$$P_{V,P} = P_{S,P} e^{\frac{P_w V_m}{R T_{m,P}}} \tag{8}$$

where $T_{m,F}$ and $T_{m,P}$ denote the membrane surface temperatures on the feed and permeate sides, respectively. Calculations of them are made on Fourier's law of heat conduction.[1]

$$T_{m,F} = T_{in} - q\left(\frac{1}{2}\frac{\delta_w}{k_w} + \frac{\delta_{gap,F}}{k_{gap}}\right) \tag{9}$$

$$T_{m,P} = T_{out} + q\left(\frac{\delta_{gap,P}}{k_{gap}} + \frac{1}{2}\frac{\delta_w}{k_w}\right) \tag{10}$$

where δ_w, $\delta_{gap,F}$, $\delta_{gap,P}$, k_w, and k_{gap} represent the thicknesses of the working plate, the liquid chamber on the feed side and the permeation side, as well as the thermal conductivities of the working plate and the liquid chamber, respectively. T_{in} and T_{out} are the temperatures of the working plate at the fluid inlet and outlet side,

respectively. The T_{in} at the first stage and the T_{out} at the final stage are the heating and cooling temperature of the system, respectively.

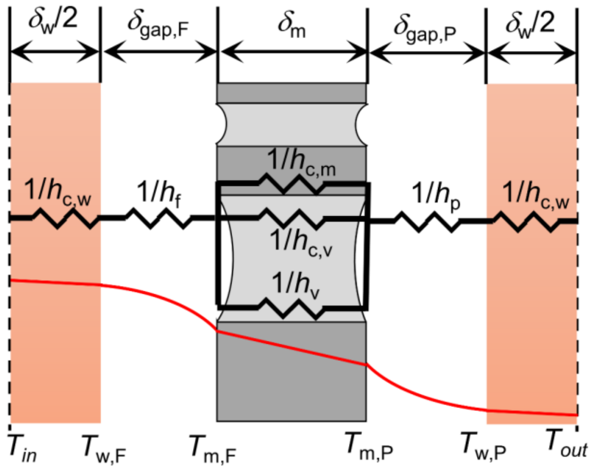

Supplementary Figure 1 Schematic diagram of the system heat transfer coefficient

Calculations will be used to determine the inside temperature, as illustrated in **Figure S1**. The heat transfer coefficients of the working plate, feed chamber, membrane, working fluid's vapor, and permeation chamber are denoted by the letters $h_{c,w}$, h_f, $h_{c,m}$, h_v, and h_p.

In Eq. (11), h_v represents the heat transfer resulting from the working fluid's phase change. It is equal to the product of the transmembrane mass flux and the evaporation enthalpy of the working fluid.[3]

$$h_v = J_w \Delta H_{vap} \tag{11}$$

The values of the above parameters are displayed in **Table S1** for calculations.

Supplementary Table 1. Calculation parameters for the prediction and experiment

	Unit	Prediction	Experiment
δ_{plate},	mm	10.0	7.5
$\delta_{gap,f}$	mm	0.1	0.15[a)]
$\delta_{gap,p}$	mm	0.2	0.30

k_{plate}	W/(m·K)	200[b)]	397
k_{gap}	W/(m·K)	0.6542	0.6542
k_{PTFE}	W/(m·K)	0.256	0.256
k_{PP}	W/(m·K)	0.22	0.22
k_{air}	W/(m·K)	0.029	0.029
ΔH_{vap}	kJ/kg	2358	2358

[a)]Estimated by the thickness of the woven mesh; [b)]Estimated by aluminum alloy (Al 6063).

The following assumptions are used in the theoretical prediction for NP-TOEC system:

1. The heating temperature of the lower working stage equals the cooling temperature of the upper working stage.

$$T_{in,i} = T_{out,i+1} (i=1, 2, 3, ... , n-1) \tag{12}$$

2. Each working stage has the same heat flux at a steady state

$$q_i = q_{i+1} (i=1, 2, 3, ... , n-1) \tag{13}$$

The deformation of the membrane has a significant effect on the system's performance, particularly when the working pressure is high, and thus cannot be neglected.[6] Several pieces of research have demonstrated that pressure primarily affects the functional layer of small pores.[7] Therefore, the compression of the functional layer is taken into consideration in this study. It is assumed that the stress and strain are linearly varying (5Mpa corresponds to 50% of the strain) within the existing range[7], and the pore size is proportional to the 1/3 power of the total pore volume in the membrane.

The heat flux of the heating surface would be higher than the theoretical prediction during the actual operation process. Because some heat may dissipate to the environment and others may conduct through the sealing ring without working. In the experiment, the heat leakage is more severe because of a relatively large silicone

ring area. However, the heat conduction through the sealing ring will be significantly reduced when the area of the sealing ring is relatively small enough in a large-scale device. According to the theoretical calculation with a heating temperature of 80 °C and no negative pressure, the heating power of the system is around 44.2 W, which only accounts for 66.2% of the total heating power. The liquid supply channel region is substantially thicker. It accounts for approximately 20% of the total heat exchange area, making it impossible to ignore its detrimental effects on heat and mass transfer resistance. In a previous study, this impact was considered by multiplying a correction factor of 0.9 by the theoretical prediction value. The CFD simulation also validated the validity of this procedure.

2. Experiment details

The membrane is a commercial PTFE filter membrane (normal pore size 0.1 μm, Xuanda). Using the static sessile drop method, a drop shape analyzer (DSA30, Kruss) assessed the membrane's contact angle. The surface morphology was examined by an environmental scanning electron microscope (Quanta 200, FEI) and a field emission scanning electron microscope (Sirion 200, FEI).[1]

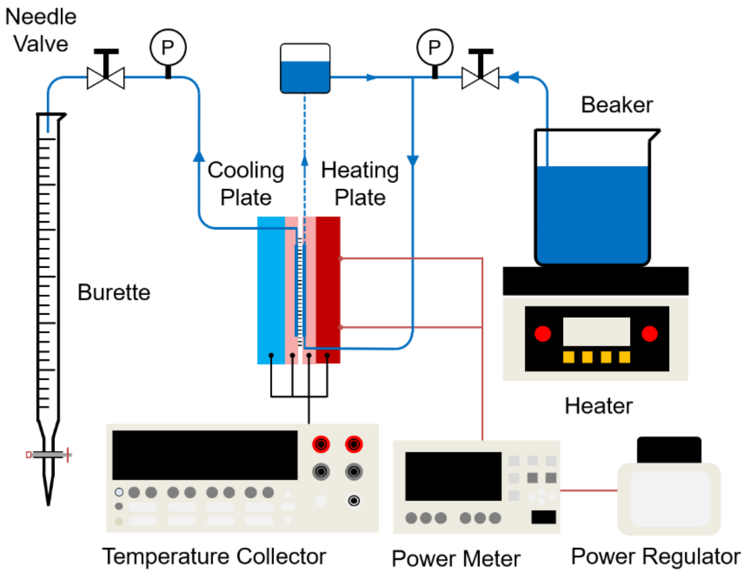

Supplementary Figure 2 Schematic diagram of the lab-scale experimental system

The experimental and data-collecting parts are the two components that make up the test system, as depicted in **Figure S2**. Before each experiment, the beaker was filled with distilled water and boiled for more than 10 minutes to remove as much noncondensable gas as possible. The polyurethane pipeline is used to transport water. The pipeline linked to the feed side has a precision regulating valve to create a specified degree of negative pressure. The pipeline connected to the permeation side also has a precision regulating valve to replicate the pressure drop of the turbine. The pressure gauge shows the pressure value (accuracy class: 2.5%). A higher precision burette rather than a graduated cylinder is employed at the output to measure the water volume for greater accuracy (accuracy: 0.05 ml). By placing a gas accumulator at the system's highest point, noncondensable gas in the cavity on the feed side is automatically collected using the difference in gas-liquid density to ensure that the gas can be discharged effectively.

On the surface of the heating plate, 1 mm deep channels are created and processed to guarantee the supply of liquid and the exclusion of noncondensable gases. The liquid layer is thicker and more resistant to heat and mass transfer where these channels are situated. Given that the abovementioned theoretical prediction is a one-dimensional ideal, the results of the theoretical performance prediction are multiplied by a correction coefficient to compare with the experimental findings based on the research of Li et al.

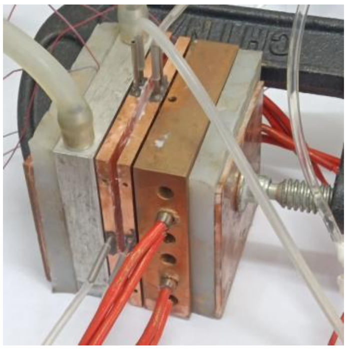

Supplementary Figure 3 Photograph of the lab-scale single-stage experimental device

The T-type thermocouple and data acquisition instrument (2700, KEITHLEY) were used to measure the cold plate and hot plate temperatures of the TOEC components; the power regulator (TDGC2-2KVA) and power meter (PF9800, EVERFINE) were used to control the hot plate temperature; and the precision regulating valve was used to control the flow rate and temperature of the water-cooling plate.

3. CFD simulation details

As shown in **Figure S4**, due to the low flow velocity in the chamber, the laminar flow model and SIMPLE algorithm are chosen for calculation. The second-order upwind discrete format is used to calculate the pressure, momentum, and energy equations. The calculation is deemed converged when the continuity residual is less than 10^{-4} and all other residuals are less than 10^{-6}. The membrane used has three layers in the experiment but only bulk in the simulation. Therefore, the membrane properties are set as the average of the real properties of the three layers. The following list of governing equations and source terms is pertinent.

Mass Conservation Equation:[8]

$$\nabla \cdot \left(\rho \vec{v} \right) = S_{\mathrm{m}} \tag{14}$$

Momentum conservation equation:[8]

$$\nabla \cdot \left(\rho \vec{v} \vec{v} \right) = -\nabla \cdot P + \nabla \cdot (\overline{\overline{\tau}}) + \rho g + S_{\mathrm{v}} \tag{15}$$

Energy conservation equation:[8]

$$\nabla \cdot \left(\rho \vec{v} C_{\mathrm{p}} T \right) = \nabla \cdot (k_{\mathrm{eff}} \nabla T) + S_{\mathrm{h}} \tag{16}$$

Mass source term S_{m}:[9]

$$S_{\mathrm{m}} = \begin{cases} -J_{\mathrm{W}} A / V & \text{at feed-membrane interface} \\ J_{\mathrm{W}} A / V & \text{at permeat-membrane interface} \end{cases} \tag{14a}$$

Momentum source term S_{v}:[9]

$$S_{\mathrm{v}} = \begin{cases} -J_{\mathrm{W}} A u / V & \text{at feed-membrane interface} \\ J_{\mathrm{W}} A u / V & \text{at permeat-membrane interface} \end{cases} \tag{15a}$$

Energy source term S_h:[9]

$$S_h = \begin{cases} -JA\Delta H_{vap}/V & \text{at feed-membrane interface} \\ JA\Delta H_{vap}/V & \text{at permeat-membrane interface} \end{cases} \quad (16a)$$

where A is the mesh area, V is the unit volume, u is the normal velocity component on the membrane surface, and h_{fg} is the gas-liquid phase variable enthalpy of the working fluid.

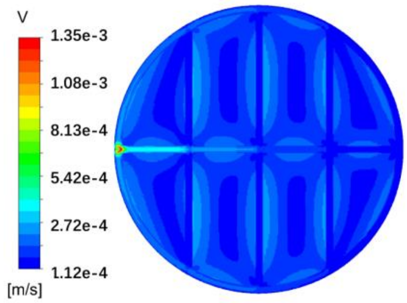

Supplementary Figure 4 Velocity contour of the fluid-membrane interface

As shown in Figure S4, except for the inlet area, the flow velocity is pretty small, about 2×10^{-4} m/s. It is worth noting that the overall flow rate of the stack TOEC system is significantly lower than that of the pump-driven DCMD system because it depends on the micro negative pressure created by the thermo-osmosis effect rather than a pump. Therefore, the contribution of convection to heat transport is minimal; only heat conduction's influence is considered.

4. Better membrane structure design

The membrane module has not been further explored and optimized for the stack TOEC system. There is still a large research space on the material, structure, hydrophobicity, mechanical strength, compressibility, etc., of the hydrophobic membrane. The major goal of this section is to optimize the insulation layer thickness for better temperature polarization.

Supplementary Table 2 Properties of different TOEC designs

	Theory design A	Theory design B	Theory design C

	Active layer	Insulation layer	Active layer	Insulation layer	Active layer	Insulation layer
Layer	1	1	1	2	1	2
Nominal pore size, μm	0.02	0.5	0.02	0.5/5	0.02	0.5/5
Porosity	0.7	0.7	0.7	0.7	0.7	0.7
Thickness, μm	10	150	10	50/100	10	50/300
Tortuosity	1.2	1.2	1.2	1.2	1.2	1.2

As shown in **Table S2**, Design A and Design B were applied to further examine the impact of lowering the mass transfer resistance of the large hole insulating layer. At the same time, the other parameters are kept the same as in Table S1, Column Prediction. The thickness of the large aperture insulation support layer in Design A is cut in half to 150 μm based on the expected membrane structure in the main text. Based on Design A, a composite structure with a 50 μm thick, 0.5 μm pore diameter and a 100 μm thick, 5 μm pore diameter is developed in design B. Due to the phenomenon that the temperature polarization coefficient decreases significantly with the mass transfer resistance[5], the film layer with a pore diameter of 5 μm is significantly thickened to 300 μm in Design C to investigate its influence on the temperature polarization coefficient and the power efficiency.

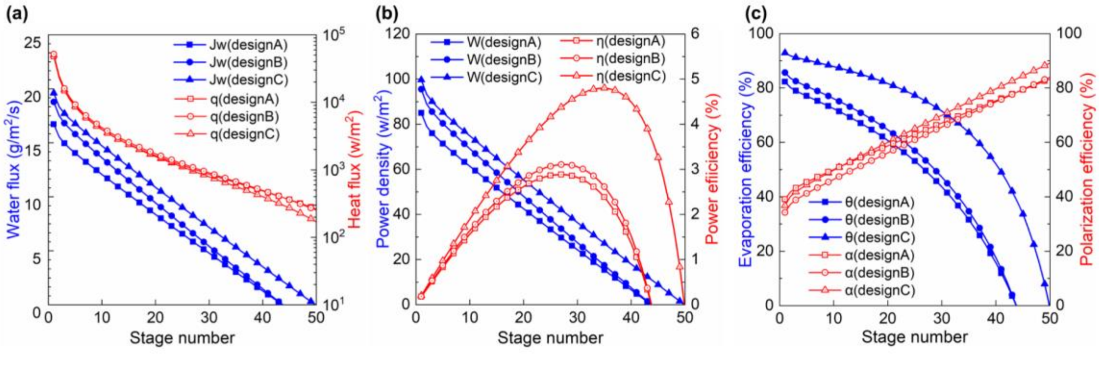

Supplementary Figure 5 Theoretical prediction performance of different membrane structures

Figure S5 shows that Design B performs slightly better than Design A overall, but the difference is not especially pronounced. This proves that in the case of complete deaeration, the low molecular mass transfer resistance limits the impact of further thinning and pore size growth of the large-pore support layer on reducing resistance. Design C, on the other hand, exhibits notable efficiency gains, with a maximum power efficiency of 4.8% at 35 working stages. Aside from evaporation efficiency, the improvement of other parameters is not immediately apparent. Therefore, raising the thickness of the membrane's macroporous layer under low mass transfer resistance may effectively increase the performance. This justifies the fabrication of pertinent membrane material and experimental study.

References

[1] J. Li, Z. Zhang, R. Zhao, B. Zhang, Y. Liang, R. Long, W. Liu, Z. Liu, *ACS Appl Mater Interfaces*. **2021**, 13, 21371.

[2] A. P. Straub, N. Y. Yip, S. H. Lin, J. Lee, M. Elimelech, *Nat Energy*. **2016**, 1, 16090.

[3] A. P. Straub, M. Elimelech, *Environ Sci Technol*. **2017**, 51, 12925.

[4] R. Long, X. Lai, Z. Liu, W. Liu, *Energy*. **2018**, 148, 1060.

[5] K. W. Lawson, D. R. Lloyd, *J Membr Sci*. **1997**, 124, 1.

[6] M. S. Lee, J. W. Chang, K. Park, D. R. Yang, *Desalination*. **2022**, 534, 115799.

[7] Z. Yuan, L. Wei, J. D. Afroze, K. Goh, Y. Chen, Y. Yu, Q. She, Y. Chen, *J Membr Sci*. **2019**, 579, 90.

[8] B. Y. Lian, Y. Wang, P. Le-Clech, V. Chen, G. Leslie, *J Membr Sci*. **2016**, 510, 489.

[9] P. Yazgan-Birgi, M. I. H. Ali, J. Swaminathan, V. J. H. Lienhard, H. A. Arafat, *J Membr Sci*. **2018**, 568, 55.
